# An Alternative Therapy for Recurrent Stasis Ulcers in Chronic Venous Insufficiency: Venocuff

**DOI:** 10.1155/2012/315147

**Published:** 2012-12-17

**Authors:** Celal Yavuz, Sinan Demirtas, Orkut Guclu, Oguz Karahan, Suleyman Yazici, Ahmet Caliskan, Binali Mavitas

**Affiliations:** Department of Cardiovascular Surgery, Medical School of Dicle University, 21280 Diyarbakir, Turkey

## Abstract

Chronic venous insufficiency may cause stasis ulcers that significantly impact on the quality of life. Many methods have been described for preventing or treating these ulcers. However, stasis ulcers often recur as a result of continuing venous insufficiency. Here we report a 30-year-old male patient with chronic venous insufficiency. He was admitted to the hospital owing to recurrent stasis ulcers. He had a history of various flavonoid drug usage and compression therapies over the previous six years. Venous Doppler sonography revealed combined saphenofemoral and deep femoral venous insufficiency. Venocuff was applied to the prejunctional and postjunctional parts of the femoral vein and the saphenofemoral junction. The patient was discharged on the postoperative second day, and a low-molecular-weight heparin dressing composed of calcium alginate was applied to the ulcer wound for one week after the operation. The stasis ulcer wound was totally healed after one month. The patient was followed up six months after the operation, and no postoperative complications or new ulceration was observed. Recurrent stasis ulcers are major reasons for hospitalization in patients with chronic venous insufficiency. Venocuff application for reducing venous insufficiency may be a good option for adjunctive ulcer therapy and for preventing recurrences of the problem.

## 1. Introduction 

Since early times, chronic venous insufficiency may have caused stasis ulcers that significantly impact the quality of life adversely. Many methods have been described for preventing or treating these ulcers. However, stasis ulcers have a tendency to recur owing to continuing venous leakage [1, 2]. Surgical methods such as high ligation, stripping, radiofrequency ablation, and endovenous laser therapy are widely performed. These are safe and effective procedures that, in experienced hands, can achieve good short- and long-term outcomes for most patients. However, the loss of the saphenous vein as a potential bypass graft and possible risk of continuous deep venous reflux are important disadvantages of these procedures [1–3]. Approaches that focus on providing venous valve sufficiency last for a long time. External wrapping is one of the available procedures in such cases. The main purpose of this approach is to restore the function of the venous valves that settle between saphenous and deep veins, by extraluminal wrapping of the dilated vein, thereby reducing its diameter and bringing the valve cusps together [3]. Here we report a case of chronic venous insufficiency associated with recurrent nonhealing venous ulcers.

## 2. Case

**Figure 1:**
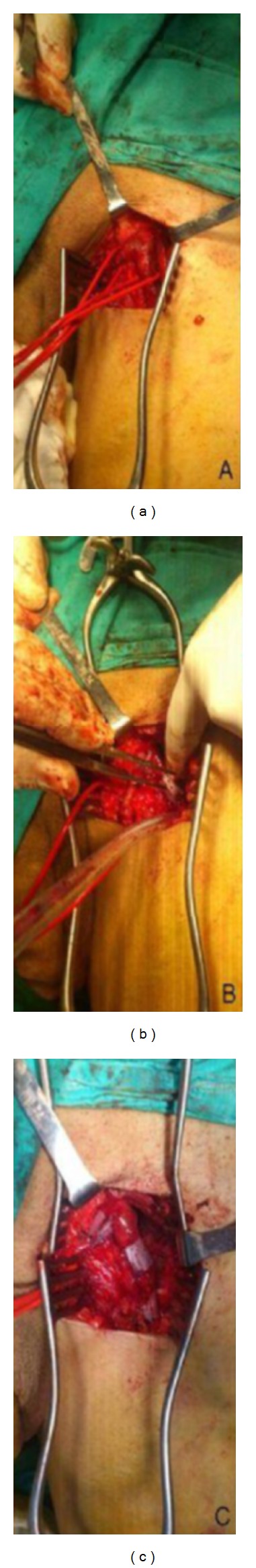
(a) Exploration of saphenofemoral venous junction. (b) Veins were turned, and Venocuff was placed around. (c) The view of the veins after the Venocuff placement.

A 30-year-old male patient with chronic venous insufficiency was admitted to hospital owing to recurrent stasis ulcers, especially on the medial side of his left tibial skin. He had a history of various flavonoid drug usage and compression therapies over the previous six years. Venous Doppler sonography revealed combined saphenofemoral and deep femoral venous leakage. Venocuff was applied to the prejunctional and postjunctional parts of femoral vein and saphenofemoral junction (Figure 1). The patient was discharged on the postoperative second day and a low-molecular-weight heparin dressing composed of calcium alginate was applied to the ulcer wound for one week after the operation. The stasis ulcer wound was totally healed after one month. The patient was followed up six months after operation, and no postoperative complications or new ulceration was observed. 

## 3. Discussion 

Chronic venous insufficiency is a common disorder worldwide and affecting one-third of the European population [4]. The impact of chronic venous insufficiency is correlated with population and socioeconomic regression owing to the loss of manpower and treatment costs [5]. 

Venous diseases are responsible for over 70% of chronic wounds in the lower extremities. Symptomatic therapies such as palliative wound healing strategies are available in current modalities. However, definitive treatment methods for recurrent venous leg ulcers are required in 72% of cases [6]. Venous leakage should be prevented, and collaborative clinical approaches must be applied in such cases.

External Venocuff strategies were suggested from many significant reports in the literature. Plentiful positive data claimed that external banding of the superficial femoral vein or saphenofemoral junction may abolish the reflux and correct venous hypertension, preventing recurrences [1, 3]. Karapolat and Ozdemir present successful applications for such cases in three patients with chronic venous insufficiency [7]. They reported this treatment to be “less invasive compared to other methods [and it] may provide an effective reconstruction in the existence of isolated valvular incompetence and reflux in saphenofemoral junction.”

In summary, recurrent stasis ulcers are major reasons for hospitalization in patients with chronic venous insufficiency. These ulcers may be treatable despite their tendency to recur. Venocuff application for reducing venous leakage may be a good option for adjunctive ulcer therapy and preventing recurrences.
